# Application of WeChat-based flipped classroom on root canal filling teaching in a preclinical endodontic course

**DOI:** 10.1186/s12909-022-03189-x

**Published:** 2022-03-02

**Authors:** Yi Zhou, Denghui Zhang, Xiaoxu Guan, Qiaoya Pan, Shuli Deng, Mengfei Yu

**Affiliations:** School of Stomatology, Stomatology Hospital, Zhejiang University School of Medicine, Zhejiang Provincial Clinical Research Center for Oral Diseases, Key Laboratory of Oral Biomedical Research of Zhejiang Province, Cancer Center of Zhejiang University, Hangzhou, 310006 China

**Keywords:** Flipped classroom, WeChat, Root canal filling, Endodontics, Root canal treatment

## Abstract

**Background:**

This study was aimed to evaluate the application of WeChat-based flipped classroom in root canal filling teaching in a preclinical endodontic course.

**Methods:**

A two‐group comparative study was designed. The pre-class test, on-site quiz, and root canal filling on extracted premolars were performed by students from a lecture-based classroom group (LG, *n* = 30) and a WeChat-based flipped classroom group (WFG, *n* = 30). Results of the Pre-class test and on-site quiz were analyzed by independent samples t-test. Post-filling radiographs were taken and evaluated by a specialist in oral radiology who was blinded to grouping. Results of root canal fillings were analyzed by the Pearson chi-square test. Student responses in questionnaires were analyzed by Fisher’s exact test.

**Results:**

The students in WFG could get significantly higher scores in the on-site test and make better performances in root canal filling than those in LG. In terms of questionnaires, students from WFG were perceived to be more motivated to learn, better to understand the knowledge, better to improve communication and clinical skills, easier to perform root canal filling but spending more time.

**Conclusion:**

The WeChat-based flipped classroom teaching can have a better effect than lecture-based teaching on root canal filling learning for students with limited endodontic experiences.

## Background

The high incidence of pulpitis and periapical periodontitis calls for an increased number of well-trained dentists to perform root canal treatment (RCT) for patients [1, 2], among which root canal filling is an important step. Endodontic education is essential not only for the training of future endodontists but also for general dentists as well. The preclinical endodontic course offers dental students a valuable chance to develop competent skills in dental clinical training [3]. However, endodontics clinical practice has a high demand for technical proficiency [4]. Lack of endodontics knowledge and poor root canal filling skills lead to increased failure of RCT and even loss of teeth in daily clinical practice [5, 6]. Furthermore, a limited understanding of root canal filling will demotivate dental students to pursue endodontics as their subspecialty after graduation [7]. Therefore, there is an increasing concern for the general quality of endodontics education, and a reform of current lecture-based teaching methods is needed to better prepare students for future careers in clinical practice [6].

The most effective methods in improving teaching efficiency are to motivate positive learning, requiring students to positively participate in the class, engage with learning materials and cooperate with classmates [8]. The flipped classroom, a new teaching model, has aroused more attention around the educational world, since it’s based on the "student-centered" curriculum design and can effectively improve students’ learning initiative [9–11].

In China, WeChat, a free application, is the most popular social networking platform with high convenience and accessibility, transmitting abundant information by video and graphics and providing a feasible way to spread medical knowledge for the students [12]. The widespread use of WeChat, especially among the students, offers an opportunity to remove the time and space obstacles in a traditional classroom. Many studies show that WeChat has great potential in medical education [13–15]. However, to our knowledge, no prior studies have focused on the combination of a WeChat-based flipped classroom and endodontics teaching.

This article is a study protocol for a randomized controlled trial study comparing a WeChat-based flipped classroom with a lecture-based classroom in root canal filling teaching. Thus, the null hypothesis is that there is no association between the flipped classroom and the quality of root canal filling acquired by undergraduate dental students in a preclinical endodontic course.

## Method

### IRB Approval

Informed consent was obtained from all subjects. The study protocol was approved by the local Ethics Committee and registered in the Chinese Clinical Trials Registry (No. ChiCTR2100044844).

### Participants

Participants were recruited from the population of year four undergraduate dental students taking a preclinical endodontic course from a dental school. The sample size was calculated using the G*Power software by Franz Faul [1]. The sample size was determined to be 28 for each group when α = 0.05, 1-β = 0.9, and effect size = 0.8, which can yield the actual power of 0.91. To achieve the sample size, included in the study were 60 students who had already been studying dentistry at the same school for 4 years but had not undergone any clinical practice in dentistry, thus having no experience in root canal filling. The total cumulative years that these undergraduate dental students have to undergo before graduation is 5 years.

These participants were randomly allocated into either the WeChat-based flipped classroom group (WFG, *n* = 30) or the lecture-based classroom group (LG, *n* = 30). The random assignment scheme was created from a table of random numbers. All students didn’t know their group assignments before the study began. Both groups had one same professor in teaching undergraduate dental students for 15 years and five same teaching assistants in supervising undergraduate dental students for a minimum period of 5 years, who work in the same dental school.

### Curriculum description

To evaluate knowledge acquisition at baseline, all students allocated in 2 groups finished the pre-class test relevant to root canal filling composed of 20 multiple choice questions each with 5 points.

**Fig. 1:**
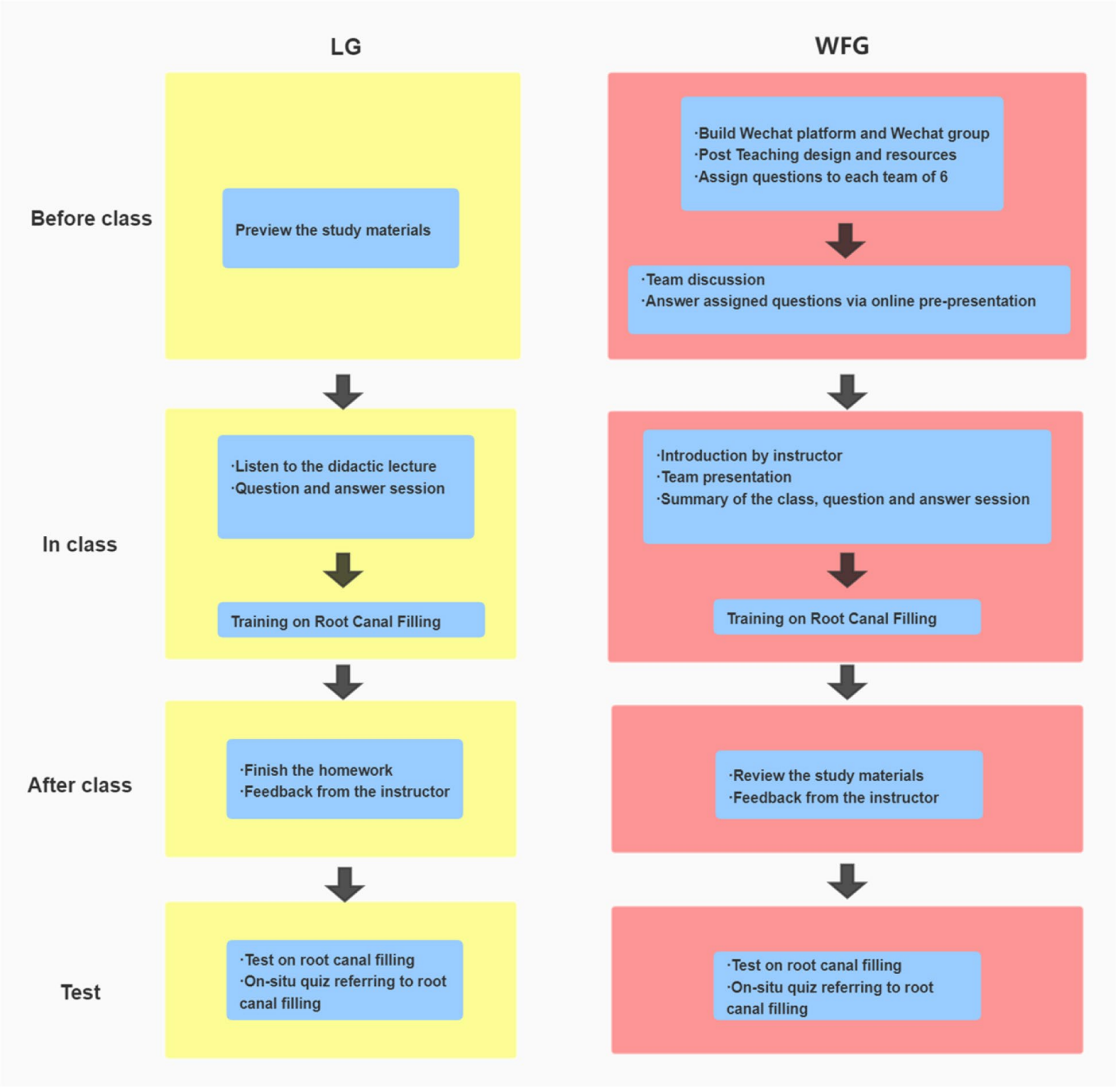
Flow diagram showing the WeChat-based flipped classroom and lecture-based classroom models. WFG: WeChat-based flipped classroom group, LG: lecture-based classroom group

To take advantage of the interactivity of WeChat and encourage collaboration of students, students in WFG were further divided into 5 small teams, with 6 students per team and each small group established its own WeChat discussion group with a teaching assistant respectively. Before the classroom session, the course materials were prepared by the professor, including supplementary study materials, a recorded practice video, and some relevant questions. Representative questions included were as followed: (1) What is the timing of root canal filling? (2) How do you determine the length of root canal filling? (3) How should the ideal root canal filling be? Fig. 1 showed the WeChat-based flipped classroom and lecture-based classroom models.

Preview of the practice video, preparation for the given questions, and a Powerpoint presentation was required before the class session. The flipped classroom began with the introduction of root canal filling and an explanation of the class agenda by the professor. Then, one student representative from each small group made a presentation to present relevant topics. The representatives also came up with the unsolved questions from each small group. After that, each small group consulted the literature and offered their answers. The teaching assistants evaluated and supplemented the answers. Then the professor summarized and answered the tough questions. After reviewing the practice video of root canal filling, a 30-min hand-on practice on 2 extracted premolars was followed. The students were encouraged to review the study materials after class to prepare for the test one week later. The time of the class including root canal filling practice was 90 min in total for students from WFG.

In the traditional lecture-based classroom, students attended a didactic lecture offline taught by the professor. Then, the same practice video was played in class followed by 30-min hand-on practice on 2 extracted premolars. After the class, the professor assigned homework including the same questions discussed in the WeChat-based flipped classroom. The students were required to complete the questions within two days. The teaching assistants corrected the homework, provided the answers online, and helped students to work out challenging questions at the students’ request. As a control, the students in LG could access the same practice video and supplementary materials as those in WFG. Also, the students were encouraged to review the study materials after class to prepare for the test one week later. The time of the class including root canal filling practice was also 90 min in total for students in LG.

The testing session, held on one week after the class session, was applied to assess the participants’ proficiency in filling root canals and examine that the students from which group could get better scores. Each student from two groups was asked to fill the canals of one extracted premolar and finish the same on-site quiz referring to root canal filling composed of 20 multiple choice questions each with 5 points. The images of all the filled premolars were screened with photostimulable phosphor plates by a periapical film machine (Sirona, Germany).

At the completion of the course, the participants in both groups completed a questionnaire modified from Paul Ramsden’s Course Experience Questionnaire and Biggs’ Study Process questionnaire [16, 17] recording their perceptions and self-assess competence.

### Specimen preparation and root filling techniques

Only mandibular premolar with one root was used in the training and test session, which was determined by visual methods and X-ray images. The access cavities of all extracted premolars included were prepared with sterile high-speed diamond burs under irrigation with saline by an experienced endodontist. All roots were shaped also by the experienced endodontist using ProTaper Universal rotary instruments (Dentsply Tulsa Dental Specialties) in the sequence recommended by the manufacturer. According to the anatomy of the root canal, apical enlargement was prepared to size F3. Each root was thoroughly irrigated during preparation with 8 mL of 3% NaOCl, rinsed with 3 mL of 17% EDTA for one minute, then flushed with 3% NaOCl for 10 s, and dried with paper points of suitable size [18]. During the preparation, working length (WL) was determined as 0.5 mm to the anatomic apical foramen. After shaping the overall canal to a tapered form, K-files of #8 or #10 should be gently moved toward the WL to assess the apical size at that point during apical gauging, and the binding point should be noted. The presence of an apical narrowing would translate into the fact that the WL might be reached without shaping effort with the desired size during gauging. However, the next larger size should stay back by a small distance. Finally, it should be verified that patency is maintained [18]. And the root canals were tried with size F3 gutta-percha cones (Dentsply Tulsa Dental Specialties) according to the size of the apical enlargement. Before root filling training or test, the working length and final diameter were informed to the students.

Students from two groups were trained in the use of warm vertical compaction technique as described according to Hargreaves et al. [18]. In brief, canals were obturated with gutta-percha cones (GAPADENT, China) and resin sealer (AHPlus, Dentsply Maillefer, America). Suitable cones with sealer were selected and fitted the prepared length. Heated pluggers (SybronEndo SE1-1, KAVO, Germany) were used to heat the master cone and remove excess coronal material. A plugger (YG21030004A, B/L, Korea) was used to compact the heated gutta-percha. And apical compaction was completed. Then a gutta-percha was injected using the battery-powered filling handle (SybronEndo SE1-2, KAVO, Germany). The heated segment was compacted by the plugger.

### Evaluation on root canal filling

The filling length and void presence could be closely related to the success rate of RCT [19], so the quality of the root canal was evaluated according to the presence of voids in the root filling materials and the distance between the radiographic apex and end of root filling in our study. Root canal treatments were grouped according to a previously published method [20] as follows: 1. Acceptable: The filling material ends 0 to 2 mm short of the radiographic apex with no voids visible, no overfilling, and with the absence of any procedural error. 2. Unacceptable: A. Under-filled: The filling material ends more than 2 mm from the radiographic apex. B. Over-filled: Materials extruded beyond the apex. C. Voids presence (no adequate density): Voids are visible within or between the material and the root canal walls. X-ray beam crossed the teeth in mesio-distal direction to get the radiographic images.

All images were evaluated by three independent specialists in endodontists in a darkened quiet room on a computer screen with Kappa > 0.8, who were all blinded to the groups. One viewing session was limited to thirty minutes to ensure reliability. If disagreement occurred, images were re-evaluated by the 3 specialists until reaching a consensus.

### Data analysis

The following results were measured: (1) Length of root filling, (2) presence of voids, (3) scores of pre-class test and on-site quiz (4) participants’ perceptions and self-assess competence in response to the questionnaires.

Data were managed and analyzed with SPSS 22.0 (SPSS, Chicago, IL). The length of root filling (adequate or inadequate), presence of voids (presence or absence), and students’ answers to the questionnaire in two groups were compared using the chi-square test. The scores of pre-class tests and on-site quiz were compared between the two groups by an independent samples t-test. Significance was set at the *P* < 0.05 level.

## Results

**Fig. 2:**
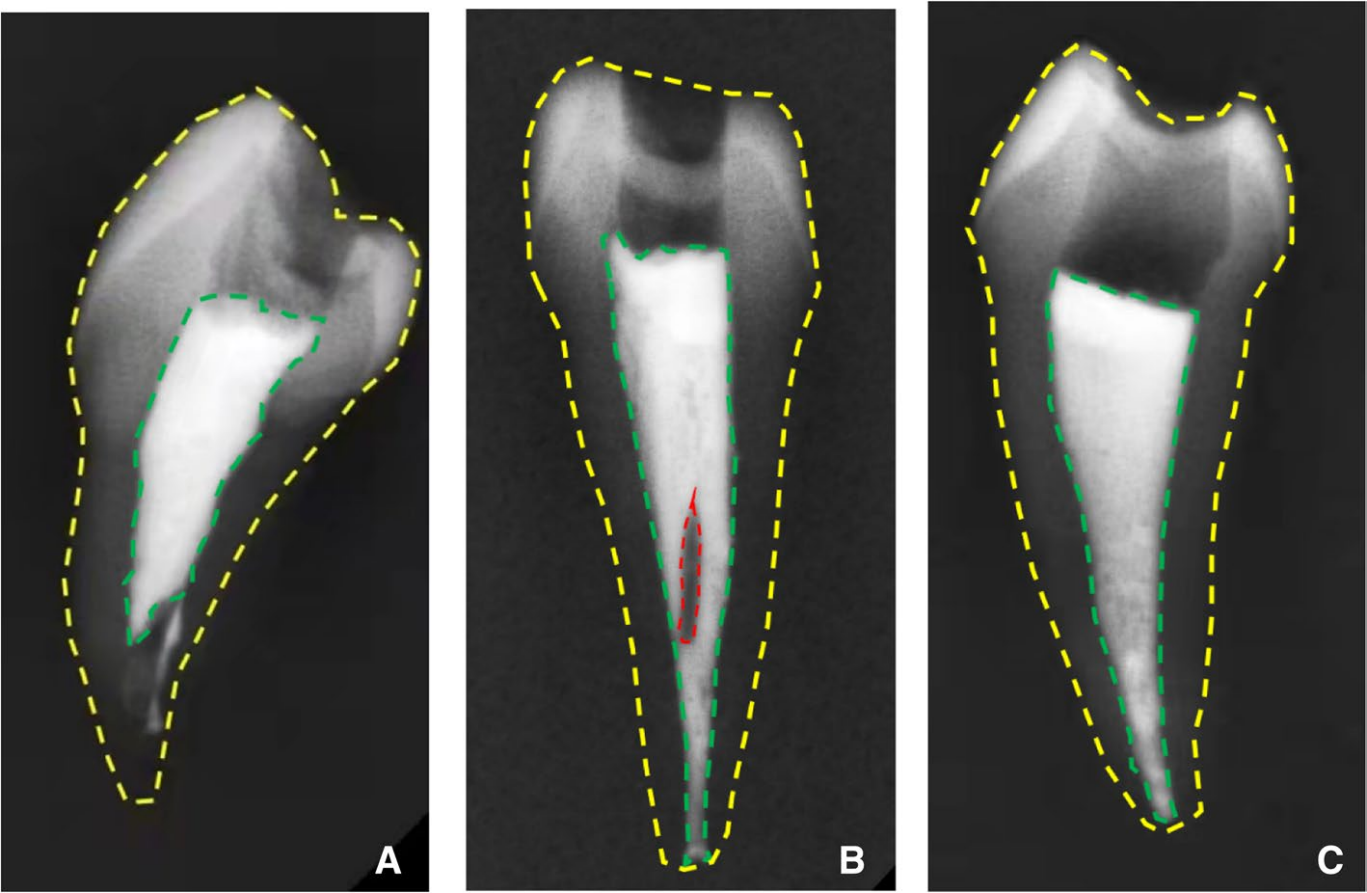
X-ray images of three typical samples. The tooth profile was outlined by a yellow dotted line. The root filling was outlined by a green dotted line. And the void was outlined by a red dotted line. **A** A root canal filled by a student from LG in test was considered unaccepted in terms of filling length. **B** A root canal filled by a student from LG in test was considered unaccepted due to void presence. **C** A root canal filled by a student from WFG in test was considered to have adequate root length and no void

A total of 60 students participated in the study, including 30 students assigned to LG and 30 students assigned to WFG. The models of LG and WFG were shown in Fig. 1. The sex ratio and ages for the two groups were comparable (Table 1). The attendance rates of class and test sessions were 100% in both groups. All students in the WFG previewed the supplementary study materials and learned the practice video of root canal filling assigned by the teaching assistants. All students in LG finished and posted the homework to the teaching assistants on time. All students finished the root canal filling and on-site quiz in the test session and all the filled premolars were screened to get the radiograph images and evaluated according to root length and void presence (Fig. 2). All students finished the questionnaires correctly.

**Table 1:** Demographic information of dental students participating in root canal filling learning

	LG	WFG	Statistics	df	*P* value
Students number	30	30			
Gender					0.796^a^
Male	15(50%)	16(53.3%)	χ2 = 0.067	1	
Female	15(50%)	14(46.7%)			
Age	21.46 ± 0.46	21.65 ± 0.50	t = 1.57	58	0.122^b^

*LG* Lecture-based classroom group, *WFG* WeChat-based flipped classroom group, *df* degrees of freedom

^a^The two groups were compared using the Pearson chi-square test

^b^The two groups were compared using Independent samples t test

Table 2 showed there was no significant difference in the two groups in regard to their pre-class test score (51.00 ± 15.39 vs. 47.67 ± 14.66, *P* = 0.394, F = 1.102), indicating that the baseline knowledge acquisition of root canal filling was comparable. However, students from WFG had significantly higher scores than students from LG (72.33 ± 11.94 vs 80.17 ± 11.78, *P* = 0.0132, F = 1.028) in the on-site quiz, indicating students from WFG had a better command of the knowledge about root canal filling than those from LG.

**Table 2:** Comparison of pre-class test and on-site quiz scores between LG and WFG

	LG	WFG	*F*	*P* value for *F* test	*t*	*P* value for *t* test
Pre-class test	51.00 ± 15.39	47.67 ± 14.66	1.102	0.796	0.859	0.394
On-site quiz	72.33 ± 11.94	80.17 ± 11.78	1.028	0.941	2.558	0.0132

*LG* Lecture-based classroom group, *WFG* WeChat-based flipped classroom group. The two groups were compared using independent samples t test. F test was used to compare variance

Table 3 showed the comparison between WFG and LG on root filling length and void presence. Among teeth filled by students, 60.0% (*n* = 18) had root fillings of acceptable length from WFG, while only 33.3% (*n* = 10) showed acceptable filling length from LG (χ2 = 4.286, *P* = 0.0384). Additionally, voids were detected in 80.0% teeth (*n* = 24) filled by students from WFG, while 93.3% teeth (*n* = 28) filled by students from LG (χ2 = 2.308, *P* = 0.1287). In total, 13.3% (*n* = 4) students had acceptable root canal filling from WFG, while only 3.3% (*n* = 1) students had acceptable root canal filling from LG (χ2 = 1.964, *P* = 0.1611).

**Table 3:** Comparison of filling length and void presence between LG and WFG

	LG	WFG	Statistics	*P* value	Effect size
Filling length					
Adequate	10 (33.3%)	18 (60.0%)			
Inadequate	20(66.7%)	12(40.0%)	χ2=4.286	0.0384	0.2679
Void presence					
Presence	28 (93.3%)	24 (80.0%)			
Absent	2 (6.7%)	6 (20.0%)	χ2 = 2.308	0.1287	0.1333

*LG* Lecture-based classroom group, *WFG* WeChat-based flipped classroom group. The two groups were compared using the Pearson chi-square test

Table 4 compared students’ perceptions after taking the WeChat-based flipped classroom and lecture-based classroom. More students from WFG felt that the course helped to improve their learning inspiration (*P* = 0.0056), understand the knowledge of root canal filling (*P* = 0.0211), and prepare for the examination (*P* = 0.0419) than those from LG. But students from WFG didn’t feel more preference on the teaching methods (*P* = 0.9999). Even more students in WFG felt that the course occupied too much spare time (*P* = 0.0056). In terms of self-assess competence, students from WFG agreed the course could improve communication skills (*P* = 0.0009), clinical thinking skills (*P* = 0.0122). Additionally, more students from WFG felt that the root canal filling had good operability through the course and test (*P* = 0.0370). However, students from WFG showed no more agreement on “I have enough time to finish root canal filling in the test” (*P* = 0.0797). These findings indicated that the WeChat-based flipped classroom method could improve students’ perceptions and self-assess competence in root canal filling teaching to some extent, but it also increased the learning burden.

**Table 4:** Comparison of students’ perceptions and self-assess competence between WFG and LG in root canal filling teaching

Questions	Group	Disagree	Agree	*P* value	Effect size
The course motivates my learning inspiration	LG	10(33.3%)	20(66.7%)	0.0056	0.3000
	WFG	1(3.2%)	29(96.7%)		
The course helps me to understand the knowledge of root canal filling	LG	10(33.3%)	20(66.7%)	0.0211	0.2667
	WFG	2(6.6%)	28(93.3%)		
The course is helpful for the final examination	LG	9(30.0%)	21(70.0%)	0.0419	0.2333
	WFG	2(6.6%)	28(93.3%)		
I like the teaching methods	LG	1(3.2%)	29(96.7%)	0.9999	0.0333
	WFG	2(6.6%)	28(93.3%)		
This course occupies too much of my spare time	LG	1(3.2%)	29(96.7%)	0.0056	0.3000
	WFG	10(33.3%)	20(66.7%)		
The course improves my communication skills	LG	14(46.7%)	16(53.3%)	0.0009	0.4000
	WFG	2(6.6%)	28(93.3%)		
The course improves my clinical thinking ability	LG	9(30.0%)	21(70.0%)	0.0122	0.2667
	WFG	1(3.2%)	29(96.7%)		
The root canal filling has good operability	LG	21(70.0%)	9(30.0%)	0.0370	0.3000
	WFG	12(40.0%)	18(60.0%)		
I have enough time to finish root canal filling in the test	LG	8(26.7%)	22(73.3%)	0.0797	0.2000
	WFG	2(6.6%)	28(93.3%)		

*LG* Lecture-based classroom group, *WFG* WeChat-based flipped classroom group. The two groups were compared using Fisher’s exact test

## Discussion

This study was an investigation into the effectiveness and suitability of the combination of a flipped classroom with the WeChat platform in root canal filling teaching. In this study, we designed a new flipped classroom mode based on WeChat applied in endodontics education. This kind of online flipped classroom successfully removed the temporal and physical limitations of the traditional flipped classroom in endodontics education. In turn, it saved more time for the quality of flipped classroom, broadened access to knowledge, and promoted efficiency in asking or solving problems in the root canal filling learning [21]. One of the most important advantages of a WeChat-based flipped classroom relies on convenience and time-saving. The traditional flipped classroom is time-consuming [22] since much time is required to gather the students to inform class schedule and on-site group discussion. However, the WeChat-based flipped classroom allows the above process to occur anytime and anywhere. As the COVID-19 has changed the whole scenario of education, different technology approaches to improve the dissemination of education are important to overcome conditions like the COVID-19 [23]. Additionally, an abundant resource could be uploaded like images, videos, and radiographic examinations via mobile phone in a timely manner. And other group members could access the resource instantly.

Multiple factors could result in the improvement of students’ performance in the WeChat-based flipped classroom method in root canal filling teaching. First, the flipped classroom approach offered personalized study [21]. Flipped classroom transformed teachers from knowledge imparter in traditional classrooms to facilitators and instructors of flipped classroom [24, 25]. Additionally, students could choose their own learning methods (video or image) and enjoy more learning freedom out of class [26]. It might account for the fact that students from WFG showed significantly higher motivation for endodontic learning in this study. In the flipped classroom approach, students mainly reinforced the transformation and absorption of knowledge through communication and discussion with teachers and classmates [27]. Furthermore, flipped classroom can be a solution for psychomotor skill [28] to some extent. In line with our study, previous studies showed that flipped classroom can effectively improve students’ knowledge and enthusiasm for learning [10]. Thus, the null hypothesis of this study was rejected.

Different from lecture-based classroom focusing on how much knowledge could be input to students, the flipped classroom could motivate students to output acquired knowledge and confusing questions [29], which might lead to the result that students from WFG felt improvement in communication skills and clinical thinking ability in our study. Thus, the flipped classroom could enhance interpersonal skills as previous study [30]. Compared to the lecture-based classroom where there was only teacher-student communication, the flipped classroom also encouraged not only teacher-student communication but also group interaction, improving individual students’ mastery of dental knowledge in turn. Similar results were also found in our study that more students in WFG showed a better understanding of endodontic knowledge both in students’ perceptions and test scores. Furthermore, compared to an average attention span of 10–20 min in the traditional lecture-based classroom [31], the flipped classroom could draw students’ attention longer and improving students’ cognitive power [32].

Plenty of advantages though the flipped classroom had the methods still failed to earn the preference by more students in WFG. The additional time for self-learning loaded the students with more pressure and burden [33], which might account for the result that more students felt overloaded from WFG. The pressure and burden could reduce the satisfaction as our questionnaire showed no difference in the preference with the teaching methods between the two groups. The inherent shortcomings of flipped classroom urged us to embrace WeChat to increase efficiency. However, despite the combination of WeChat and flipped classroom, students still felt the course consumed too much time. To increase the learning efficiency without overwhelming the students, the new teaching mode should be improved to fully integrate the advantages of the flipped classroom and WeChat platform. And inaccessibility to technical gadgets by a section of students and the need for more screen time might also increase the learning burden of students [33]. The teachers will also have to be trained for taking up such type of unconventional teaching [34]. Moreover, by taking advantage of the interactivity with WeChat, more learning hours might be provided for students in group WFG, thus leading to a risk of bias.

Previous studies reported some teaching problems regarding undergraduate endodontic education, related to lack of staff and teaching hours [35–37]. And in the early stages of endodontic learning, inexperienced dental students need explicit directions for performing detailed steps [38]. Therefore, performing few isolated skills under standardized conditions is an essential requirement for successful transfer from preclinical learning to clinical practice [38, 39]. Clinical steps for the RCT consist of three separate skills: access cavity preparation, canal cleaning and shaping, and root canal filling. In our study, the root canal filling technique was isolated to be taught to promote the clinical skills of dental students. the WeChat-based flipped classroom could significantly increase the rate of adequate filling length. And fewer students performed root filling with a void from WFG. The results suggested the new teaching mode could increase the learning efficiency of clinical skills and improve the root filling quality performed by novice dental students. These results implied that dental educators should take particular account for the WeChat-based flipped classroom methods in stimulating students’ learning initiative and acquiring endodontics knowledge and clinical skills.

Our study has several limitations. Firstly, assessment of the WeChat-based flipped classroom model is partly based on self-evaluation from the participators, such as clinical thinking and communication skills. Thus, the more advanced system including measures of the above competencies will be developed to better evaluate the effectiveness of the WeChat-based flipped classroom. Secondly, we have chosen only root canal filling to evaluate the effectiveness of the WeChat-based flipped classroom model. Apart from root canal filling, instrumentation of the canals is also critical to the outcomes of RCT [18]. The effectiveness of this teaching mode for all RCT procedures not only filling should be researched in further investigation. Thirdly, the study only investigated the new teaching mode applied in students in one dental school. An enlarged and multi centric trial should be performed to confirm this tendency. Fourth, this method has limitations as radiographic images provide only two-dimensional information on a three-dimensional structure and may be subject to magnification and distortion. In addition, small volumes of voids may not be visualized.

## Conclusion

The WeChat-based flipped classroom approach might be an alternative to the lecture-based classroom in the teaching of root canal filling during the preclinical endodontic course. The new approach might improve students’ clinical skills, motivate their learning enthusiasm, and enhance communication skills and clinical thinking. But the new teaching mode still requires more improvements to reduce students’ workload and gain more preference.

## Data Availability

All data are available in the main text or the supplementary materials.

## Abbreviations

LG: Lecture-based classroom group
WFG: WeChat-based flipped classroom group
RCT: Root canal therapy

## References

[1] Plasschaert AJ, Holbrook WP, Delap E, Martinez C, Walmsley AD (2005). Profile and competences for the European dentist. Eur J Dent Educ.

[2] Ilgüy D, Ilgüy M, Fisekçioglu E, Ersan N, Tanalp J, Dölekoglu S (2013). Assessment of root canal treatment outcomes performed by Turkish dental students: results after two years. J Dent Educ.

[3] Henzi D, Davis E, Jasinevicius R, Hendricson W (2007). In the students' own words: what are the strengths and weaknesses of the dental school curriculum?. J Dent Educ.

[4] Chambers DW (2001). Preliminary evidence for a general competency hypothesis. J Dent Educ.

[5] Habib AA, Doumani MD, Nassani MZ, Shamsy E, Jto BS, Arwadİ HA, Mohamed SA (2018). radiographic assessment of the quality of root canal fillings performed by senior dental students. Eur Endod J.

[6] Eleftheriadis GI, Lambrianidis TP (2005). Technical quality of root canal treatment and detection of iatrogenic errors in an undergraduate dental clinic. Int Endod J.

[7] AlRahabi MK (2017). Evaluation of complications of root canal treatment performed by undergraduate dental students. Libyan J Med.

[8] Freeman S, Eddy SL, McDonough M, Smith MK, Okoroafor N, Jordt H, Wenderoth MP (2014). Active learning increases student performance in science, engineering, and mathematics. Proc Natl Acad Sci USA.

[9] Hew KF, Lo CK (2018). Flipped classroom improves student learning in health professions education: a meta-analysis. BMC Med Educ.

[10] Bohaty BS, Redford GJ, Gadbury-Amyot CC (2016). Flipping the classroom: assessment of strategies to promote student-centered, self-directed learning in a dental school course in pediatric dentistry. J Dent Educ.

[11] Shi CR, Rana J, Burgin S (2018). Teaching & learning tips 6: the flipped classroom. Int J Dermatol.

[12] Zhang X, Wen D, Liang J, Lei J (2017). How the public uses social media wechat to obtain health information in china: a survey study. BMC Med Inform Decis Mak.

[13] Wang J, Gao F, Li J, Zhang J, Li S, Xu GT, Xu L, Chen J, Lu L (2017). The usability of WeChat as a mobile and interactive medium in student-centered medical teaching. Biochem Mol Biol Educ.

[14] Zhang W, Li ZR, Li Z (2019). WeChat as a platform for problem-based learning in a dental practical clerkship: feasibility study. J Med Internet Res.

[15] Tu S, Yan X, Jie K, Ying M, Huang C (2018). WeChat: an applicable and flexible social app software for mobile teaching. Biochem Mol Biol Educ.

[16] Ramsden P (1991). A performance indicator of teaching quality in higher education: the course experience questionnaire. Stud High Educ.

[17] Biggs J (1987). Student Approaches to Learning and Studying: Study Process Questionaire Manual.

[18] Hargreaves KM, Berman LH, Rotstein I, Cohen S (2015). Cohen’s Pathways of the Pulp: Cohen’s Pathways of the Pulp.

[19] Khabbaz MG, Protogerou E, Douka E (2010). Radiographic quality of root fillings performed by undergraduate students. Int Endod J.

[20] Kharouf N, Hemmerlé J, Haikel Y, Mancino D (2019). Technical quality of root canal filling in preclinical training at strasbourg university using two teaching protocols. Eur J Dent.

[21] Compeau P (2019). Establishing a computational biology flipped classroom. PLoS Comput Biol.

[22] Anderson HG, Frazier L, Anderson SL, Stanton R, Gillette C, Broedel-Zaugg K, Yingling K (2017). Comparison of pharmaceutical calculations learning outcomes achieved within a traditional lecture or flipped classroom andragogy. Am J Pharm Educ.

[23] Khan MSH, Abdou BO (2021). Flipped classroom: how higher education institutions (HEIs) of Bangladesh could move forward during COVID-19 pandemic. Soc Sci Humanit Open.

[24] Pan X (2020). Technology acceptance, technological self-efficacy, and attitude toward technology-based self-directed learning: learning motivation as a mediator. Front Psychol.

[25] French H, Arias-Shah A, Gisondo C, Gray MM (2020). Perspectives: the flipped classroom in graduate medical education. NeoReviews.

[26] Lin Y, Zhu Y, Chen C, Wang W, Chen T, Li T, Li Y, Liu B, Lian Y, Lu L (2017). Facing the challenges in ophthalmology clerkship teaching: is flipped classroom the answer?. PLoS One.

[27] McLaughlin JE, Roth MT, Glatt DM, Gharkholonarehe N, Davidson CA, Griffin LM, Esserman DA, Mumper RJ (2014). The flipped classroom: a course redesign to foster learning and engagement in a health professions school. Acad Med.

[28] Dinndorf-Hogenson GA, Hoover C, Berndt JL, Tollefson B, Peterson J, Laudenbach N (2019). Applying the flipped classroom model to psychomotor skill acquisition in nursing. Nurs Educ Perspect.

[29] Mortensen CJ, Nicholson AM (2015). The flipped classroom stimulates greater learning and is a modern 21st century approach to teaching today's undergraduates. J Anim Sci.

[30] Ihm J, Choi H, Roh S (2017). Flipped-learning course design and evaluation through student self-assessment in a predental science class. Korean J Med Educ.

[31] Stuart J, Rutherford RJ (1978). Medical student concentration during lectures. Lancet.

[32] Ahmed MMH, Indurkhya B (2020). Investigating cognitive holding power and equity in the flipped classroom. Heliyon.

[33] Gillette C, Rudolph M, Kimble C, Rockich-Winston N, Smith L, Broedel-Zaugg K (2018). A meta-analysis of outcomes comparing flipped classroom and lecture. Am J Pharm Educ.

[34] Gómez-Carrasco CJ, Monteagudo-Fernández J, Moreno-Vera JR, Sainz-Gómez M (2020). Evaluation of a gamification and flipped-classroom program used in teacher training: perception of learning and outcome. PLoS One.

[35] Barrieshi-Nusair KM, Al-Omari MA, Al-Hiyasat AS (2004). Radiographic technical quality of root canal treatment performed by dental students at the dental teaching center in Jordan. J Dent.

[36] Dummer PM (1991). Comparison of undergraduate endodontic teaching programmes in the United Kingdom and in some dental schools in Europe and the United States. Int Endod J.

[37] Qualtrough AJ, Dummer PM (1997). Undergraduate endodontic teaching in the United Kingdom: an update. Int Endod J.

[38] Hauser AM, Bowen DM (2009). Primer on preclinical instruction and evaluation. J Dent Educ.

[39] Chambers DW (1987). Issues in transferring preclinical skill learning to the clinical context. J Dent Educ.

